# Isolated Pectoralis Major and Tricep Atrophy Secondary to Neuralgic Amyotrophy

**DOI:** 10.7759/cureus.83599

**Published:** 2025-05-06

**Authors:** Robin Mata, Emma M Eng, Christopher Alessia, Xavier Aviles, Angie Lastra

**Affiliations:** 1 Physical Medicine and Rehabilitation, University of Miami/Jackson Memorial Hospital, Miami, USA; 2 Physical Medicine and Rehabilitation, Florida Atlantic University Charles E. Schmidt College of Medicine, Boca Raton, USA; 3 Physical Medicine and Rehabilitation, Bruce Carter Miami Veterans Administration Hospital, Miami, USA

**Keywords:** brachial neuritis, immune brachial plexopathy, lateral pectoral nerve neuropathy, massage, muscular atrophy, neuralgic amyotrophy, parsonnage-turner syndrome, pectoralis major atrophy, radial neuropathy, triceps atrophy

## Abstract

Neuralgic amyotrophy (NA), or Parsonage-Turner syndrome, describes a spectrum of relatively rare peripheral neuropathies characterized by acute pain followed by patchy, multifocal sensory disturbances, weakness, and atrophy, predominantly in the upper extremity. While typically affecting the upper brachial plexus, NA can also present with selective involvement of distal nerve branches. This case report details a unique presentation of NA in a 39-year-old male bodybuilder who developed isolated weakness and atrophy of the left pectoralis major and a single head of the left triceps following a painful prodrome without preceding trauma. Electrodiagnostic studies confirmed active denervation in these muscles, while imaging revealed no cervical pathology or brachial plexus abnormalities. The patient was diagnosed with NA, specifically affecting the lateral pectoral nerve, and a branch of the radial nerve.

Our case is compared to existing literature, including isolated reports of pectoralis major and branch-specific radial nerve involvement in NA. While these less common deficits have been described as separate entities, our case uniquely demonstrates them co-occurring. It highlights the potential for NA to affect less common nerves (lateral pectoral and motor branches of the radial nerve) and specific nerve branches.

Differential diagnoses included mononeuritis multiplex, compressive neuropathy, and infectious or inflammatory neuropathies. Mononeuritis multiplex was less likely due to the absence of sensory deficits and systemic symptoms. Infectious and inflammatory neuropathies were ruled out based on clinical presentation and lack of relevant markers. Compressive neuropathy was considered, however the acute painful prodrome, electrodiagnostic findings, and natural history favored NA.

This case contributes to understanding the variable clinical spectrum of NA. It also underscores the importance of clinical history and comprehensive work-up in atypical presentations for prompt diagnosis and effective management. While the prognosis for NA is generally favorable, the long-term outcome for highly selective presentations requires further investigation.

## Introduction

Neuralgic amyotrophy (NA), also known as Parsonage-Turner syndrome, is a rare peripheral nerve disorder characterized by acute, severe pain in the shoulder girdle followed by rapid onset of sensory disturbances, multifocal motor weakness and atrophy, primarily affecting the upper extremity with or without phrenic nerve and lumbosacral involvement [1]. The condition presents in idiopathic (INA) and hereditary (HNA) forms. The estimated incidence of INA ranges from two to four cases per 100,000 people annually, with HNA expected to be 10 times rarer than INA [2,3]. Overall, underdiagnosis of NA is likely due to its variable presentation and overlap with other neuromuscular conditions [2,3]. Men are affected more frequently than women (male-to-female ratio approximately 1.5-3:1), with peak onset typically between the third and fifth decades of life, though HNA may present earlier in the second decade [2,4].

The prodromal phase of NA is often preceded by triggers such as infections (e.g., viral, bacterial), vaccinations, surgery, or trauma. Preceding immunological events have been reported in up to 50% of cases (previously listed as well as pregnancy, childbirth, and immunotherapy). Unusually strenuous physical exercise has been reported in approximately 10% of cases [5]. These triggers are followed by the hallmark of acute pain, typically unilateral, which is severe (numeric rating scale ≥7), and localized to the shoulder, neck, or arm [1]. This pain, described as burning or stabbing, usually resolves within days to weeks. It is followed by patchy sensory symptoms, weakness, and atrophy in muscles innervated by the brachial plexus or its branches, such as the deltoid, biceps brachii, triceps brachii, pectoralis major, serratus anterior, and infraspinatus [5,6]. One hypothesis suggests that the pre­dilection of neuralgic amyotrophy for the brachial plexus (especially the upper trunk, the most mobile part) is mediated by wear-and-tear-induced weakening of the blood-nerve barrier that normally prevents any soluble immune factors or cells from coming into contact with the peripheral nervous system [5].

The long thoracic nerve is most commonly affected of the named peripheral nerves, followed by the suprascapular, anterior interosseous, lateral antebrachial cutaneous, and/or the superficial radial sensory nerves [5]. Muscle atrophy is a common sequela [5]. Two notable case reports by Parnes et al. and Zara et al. document less commonly involved nerves in NA, like the lateral pectoral and branch-specific radial motor nerve [7,8]. Parnes et al.’s case describes mononeuropathy of the lateral pectoral nerve with pectoralis major atrophy of the clavicular portion [7]. Zara et al. reported minimal strength deficit of the triceps muscle without notable atrophy but partial active denervation of the triceps on electromyography (EMG) [8]. 

Diagnosis of NA relies on clinical history, physical examination, imaging, and electrodiagnostic studies, particularly EMG, which excludes other causes and reveals a neurogenic pattern of patchy denervation in affected muscles [4]. High-resolution magnetic resonance imaging (MRI) and ultrasound have emerged as valuable adjuncts, identifying pathognomonic hourglass-like constrictions in affected nerves, enhancing diagnostic accuracy [9]. Differential diagnoses must be excluded, including cervical radiculopathy, rotator cuff pathology, and amyotrophic lateral sclerosis [3].

Prognosis for recovery varies with the amount of axonal damage, serving as a fair prediction of the possibility of nerve recovery [5]. Ranges for recovery differ significantly. Many studies report recovery to ~80-90% of previous health after two to three years in a majority of patients [5], conflicting reports state that >70% are left with some residual paresis and exercise intolerance, especially of the periscapular muscles [5,10]. Other studies indicate persistent pain, debility, and fatigue can range from 25-80% among those afflicted [10]. Collateral reinnervation will likely be incomplete if >70% of axons are damaged [5]. Clinically, this is comparable to initial paresis of grade ≤3 on muscle strength testing [5]. Incomplete collateral reinnervation makes further recovery dependent on proximal reinnervation, which can take several years and may be incomplete [5]. Other factors such as preceding trauma, younger age at initial occurrence, unilateral involvement, and limited muscles affected may potentially predict a greater chance of spontaneous recovery [11]. 

Treatment is primarily supportive, focusing on pain management with analgesics and tailored physical therapy [12]. Anecdotally, acute pain management of NA may include a course of corticosteroids, nonsteroidal anti-inflammatory drugs (NSAIDs), neuropathic pain agents, and/or short courses of opioid medications for severe cases. To date, no randomized controlled trials exist to provide evidence of superiority [2]. Corticosteroids and intravenous immunoglobulin (IVIG) are used off-label, with evidence suggesting benefit, particularly if initiated early in the disease course, but evidence is limited to case-series level data [7,13]. Surgical intervention, such as microneurolysis, may be considered for persistent weakness associated with hourglass-like constrictions confirmed on imaging [9].

No universally accepted guidelines exist for NA management, but expert consensus emphasizes early diagnosis, multidisciplinary care, and individualized rehabilitation to address compensatory motor patterns and fatigue [12]. The NA-CONTROL trial protocol highlights outpatient rehabilitation targeting motor control and self-management strategies to reduce pain and fatigue as a promising approach [14]. This case report aims to contribute to the literature by detailing a unique presentation of NA affecting the lateral pectoral nerve and motor branches of the radial nerve, highlighting diagnostic challenges, and discussing treatment outcomes in the context of current evidence.

## Case presentation

A 39-year-old male bodybuilder with a past medical history of chronic neck pain secondary to myofascial pain presented to the emergency department (ED) complaining of severe, persistent pain to his left upper shoulder girdle without immediate preceding trauma, focal neurologic deficits, or weakness on muscle testing. The patient had previously received trigger point injections to the cervical musculature with significant pain relief. As part of mutual decision making, he received trigger point injections with a solution of 1% lidocaine and 4 mg Decadron to various points in the left rhomboid. He was discharged home with ibuprofen, cyclobenzaprine, and a lidocaine patch. He returned to the ED four days later due to progressive intractable pain in the left shoulder girdle without new symptoms or changes in physical exam. He was then given subcutaneous Toradol and Decadron and discharged home with methocarbamol and naproxen, which led to approximately 50% improvement in his pain.

Seeking further pain relief, he underwent a deep tissue massage with a Graston tool to his left upper extremity and deep axillary region, described as intensely painful. Approximately 10 days following this massage, he developed numbness and paresthesia to the entire left first to third digits, and he noted acute painless weakness in his left triceps muscle when performing tricep extension weighted exercises. He attributed these symptoms to the recent deep tissue massage. 

**Figure 1:**
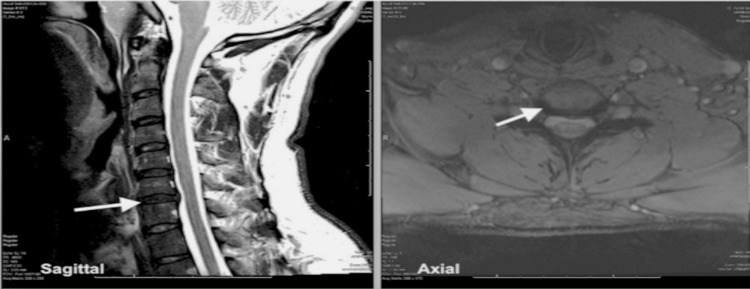
T2 Sagittal and Axial Views of the Cervical Spine and the C6-C7 level on MRI. A broad-based disc bulge at the level of C6-C7 is noted without neuroforaminal or central stenosis. No other acute pathology is noted on imaging. Left: T2 Sagittal View of Cervical Spine on MRI. Right: Axial View of Cervical Spine at the level of C6-C7 on MRI.

The patient presented to our clinic five weeks following the initial ED visit, endorsing new-onset subjective weakness in his left triceps muscle and paresthesia to his entire left first to third digits. He denied prior falls or recent trauma to his neck and upper extremities. The patient reported a 30-lb decrease in working weight on triceps extension compared to his right triceps. Initial physical exam was unremarkable for focal neurologic deficits except for numbness over the left first to third digits. The examining physician elicited a 5/5 on triceps extension during Manual Muscle Testing (MMT) comparable with the patient’s right side. To evaluate for cervical radiculopathy, cervical magnetic resonance imaging (MRI) (Figure 1) and electrodiagnostic studies were ordered; imaging revealed no significant cervical pathology. Recent labs obtained by the patient’s primary care physician, including auto-immune panel, were within normal limits. 

On his return visit over a month later, his paresthesia had resolved. New left pectoralis major atrophy of the clavicular portion and slight left triceps atrophy with 4/5 strength on MMT were observed. This prompted further work-up with brachial plexus MRI and a Neurology consult. 

Electrodiagnostic studies were performed 69 days after his initial injury, including evaluation of the pectoral nerves. Findings revealed active denervation of the left pectoralis major, isolated head of the left triceps muscle, and low F wave-persistence of the radial nerve (Table 1). Distal radial nerve innervated musculature was unaffected (Table 2). Brachial plexus MRI showed no evidence of pathology. Following review of these results, the patient underwent an MRI of the left shoulder and chest, which revealed an intact left pectoralis major tendon with focal fatty infiltration. The patient’s working diagnosis was favored to be compression-induced neuropathy vs. neuralgic amyotrophy affecting the lateral pectoral nerve and the branch of the radial nerve. Differential diagnosis included mononeuritis multiplex and infectious or inflammatory neuropathies. 

**Table 1:** Lateral Pectoral Nerve Electrodiagnostic Studies Findings: Prolonged latency and decreased amplitude in the left lateral pectoral nerve, consistent with axonotmesis. Abbreviations: Stim, stimulation; O-P Amp, Onset-Peak Amplitude

Stim Site	Onset (ms)	Normal onset (ms)	O-P Amp (mV)
Left Clavicle	1.8	<5	3.8
Right Clavicle	1.3	<5	6.2

**Table 2:** Electromyography results of the left upper extremity. Positive sharp waves in the left triceps and pectoral major indicating active denervation without evidence of active denervation in other radial, pectoral, or C5-C8 innervated musculature. Abbreviations: Ins Act, insertional activity; Fibs, fibrillations; Psw, positive sharp waves; Amp, amplitude; Dur, duration; Poly, polyphasic; Recrt, recruitment; Int Pat, interference pattern; Inc, Increased

Side	Muscle	Nerve	Root	Ins Act	Fibs	Psw	Amp	Dur	Poly	Recrt	Int Pat
Left	Abd Poll Bre	Median	C8-T1	Nml	Nml	Nml	Nml	Nml	0	Nml	Nml
Left	Biceps	Musculocut	C5-6	Nml	Nml	Nml	Nml	Nml	0	Nml	Nml
Left	Triceps	Radial	C6-7-8	Inc	Nml	2+	Nml	Nml	0	Nml	Nml
Left	Deltoid	Axillary	C5-6	Nml	Nml	Nml	Nml	Nml	0	Nml	Nml
Left	C7 Paraspinal	Rami	C7	Nml	Nml	Nml					
Left	Ext Indicis	Radial (Post Int)	C7-8	Nml	Nml	Nml	Nml	Nml	0	Nml	Nml
Left	Rhomboid Major	Dorsal Scap	C5	Nml	Nml	Nml	Nml	Nml	0	Nml	Nml
Left	Supraspinatus	SupraScap	C5-6	Nml	Nml	Nml	Nml	Nml	0	Nml	Nml
Left	Infraspinatus	SupraScap	C5-6	Nml	Nml	Nml	Nml	Nml	0	Nml	Nml
Left	LatisDorsi	ThoracoDors	C6-8	Nml	Nml	Nml	Nml	Nml	0	Nml	Nml
Left	Pect Major	Pectoral	C5-6	Inc	Nml	2+	Nml	Nml	0	Nml	Nml
Left	C6 Paraspinal	Rami	C6	Nml	Nml	Nml					
Left	BrachioRad	Radial	C5-6	Nml	Nml	Nml	Nml	Nml	0	Nml	Nml

Five months following initial presentation, he was seen by Neurology. Notable physical exam findings were 5/5 in triceps extension on MMT and persistent left pectoral atrophy at the clavicular head without focal neuro deficits. At that time, he reported subjective improvement in the strength of his triceps of 15-20%. Given his history of acute onset, lack of disease progression, imaging findings, and electrodiagnostic studies, he was diagnosed with NA, and repeat EMG was ordered to assist with prognosis. He was counseled to continue conservative management with physical therapy and regular monitoring. The patient completed several sessions of physical therapy before being lost to follow-up. 

## Discussion

NA, or Parsonage-Turner syndrome, is a multifocal neuropathy characterized by acute pain followed by weakness and atrophy, often affecting specific named peripheral nerves rather than the brachial plexus proper. This case report describes a rare presentation of NA with isolated lateral pectoral neuropathy and atrophy confined to a single head of the triceps brachii, highlighting the selective fascicular involvement characteristic of NA. The focal nature of nerve branch involvement aligns with the pathophysiology of NA, where inflammatory or mechanical processes, such as hourglass-like constrictions, target specific nerve fascicles [6]. We compare our case to two reported cases by Parnes et al. and Zara et al. [7,8], which also demonstrate less common peripheral nerve and branch-specific involvement, and discuss differential diagnoses, including mononeuritis multiplex and compressive neuropathies, to contextualize diagnostic and prognostic considerations.

Branch-specific involvement in neuralgic amyotrophy

**Figure 2:**
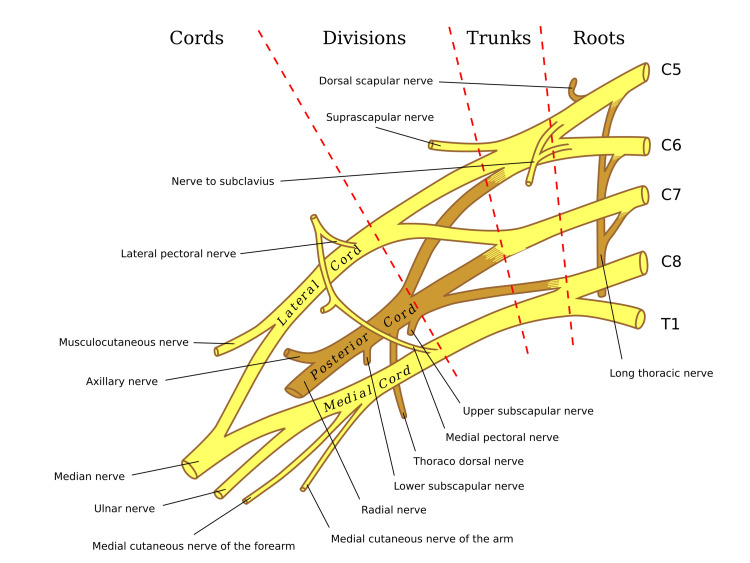
Brachial Plexus illustrating lateral pectoral nerve origin from lateral cord and radial nerve origin from posterior cord prior to innervating the triceps muscle. This image is available for use under the Creative Commons License [15]

**Figure 3:**
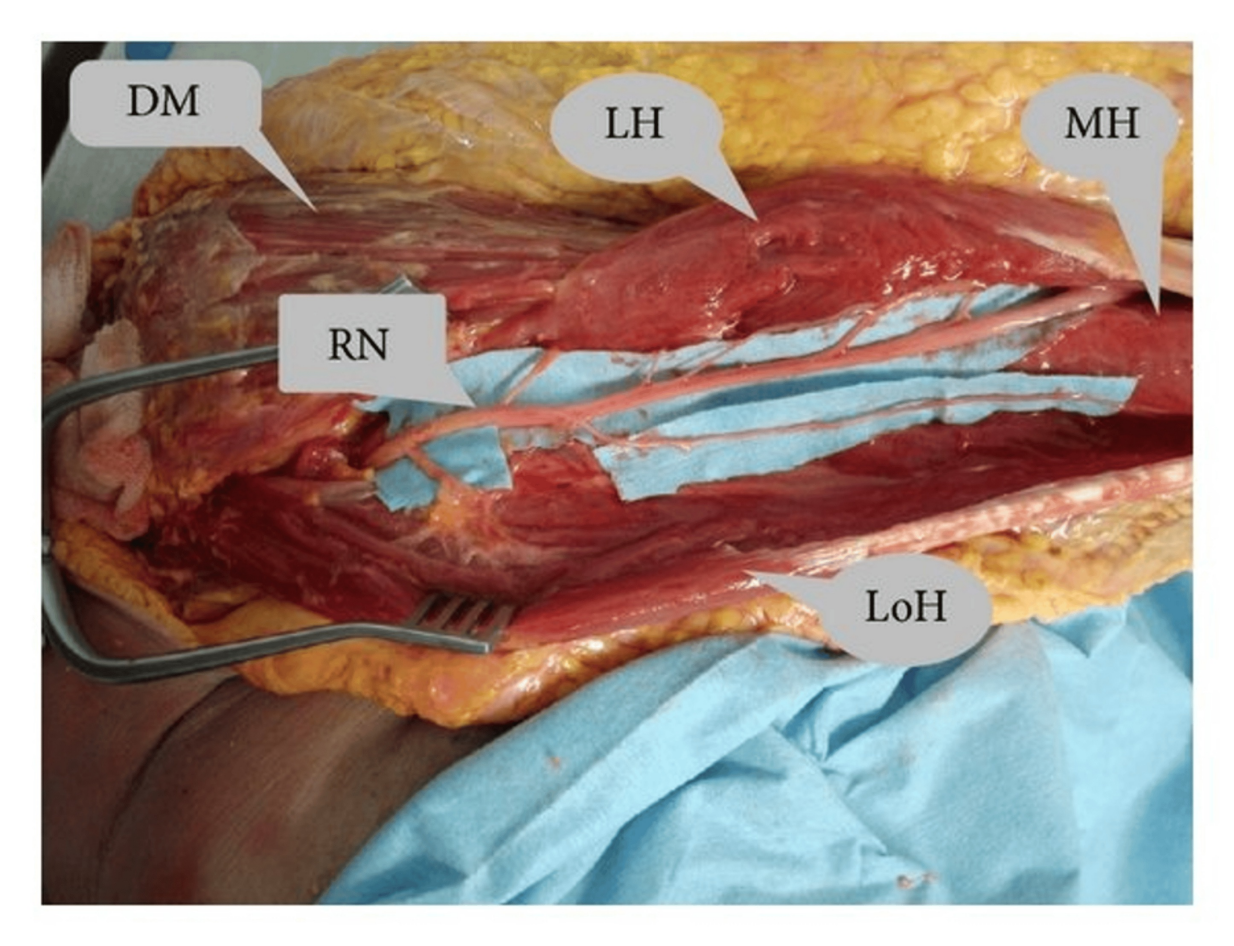
Cadaveric dissection demonstrating branches of radial nerve (RN) to long head of triceps (LoH), medial head of triceps (MH), and long head (LH). Credit: Al-Meshal and Gilbert, 2013. This image is available for use under the Creative Commons License [16]

The selective involvement of peripheral nerve branches in NA is well-documented and thought to arise from focal inflammation, immune-mediated damage, and/or mechanical torsion affecting nerve fascicles [1,9]. In our case, the patient presented with acute shoulder pain followed by weakness and atrophy limited to muscles innervated by the lateral pectoral nerve (pectoralis major) and a single head of the triceps brachii (likely the long head, innervated by a radial nerve branch) (Figures 2, 3) [15,16]. EMG confirmed denervation in these muscles, with normal findings in other muscles of radial and brachial plexus innervated distributions, supporting a diagnosis of NA. This selective pattern underscores the fascicular vulnerability in NA, where specific named peripheral nerve or their branches are targeted, potentially sparing the parent nerve. 

In comparison, Parnes et al. reported a 48-year-old male recreational weight lifter with sudden onset of pain in his right shoulder after routine workout who developed pectoralis major atrophy in the following weeks [7]. Magnetic resonance imaging of the chest revealed intramuscular edema and muscular atrophy of the pectoralis major without fatty infiltration. At three months following symptom onset, EMG revealed active denervation with reduced recruitment of the upper two-thirds of the pectoralis major consistent with mononeuropathy of the lateral pectoral nerve, likely secondary to Parsonage-Turner syndrome. Nine months after the onset of symptoms, the patient reported significant subjective improvement. Physical examination revealed decreased muscle atrophy with improved pectoralis muscle strength 4/5 on MMT. Follow-up EMG at six months demonstrated interval improvement, with full recovery reported at 18 months. Similar to our case, Parnes et al.’s patient had involvement of the pectoralis major, albeit without focal fatty infiltration on MRI, and recovery occurring at nine months post-onset [7]. However, both cases share the hallmark NA features of antecedent pain and atrophy in the pectoralis major, reinforcing the condition’s propensity for focal peripheral nerve involvement rather than nerve root distributions.

Similarly, Zara et al. described a 55-year-old woman with burning, radiating down her right arm and numbness in the first three fingers of the hand with mild triceps brachii weakness of 4/5 on MMT [8]. EMG at 13 days was unremarkable; however, one-month repeat EMG showed slight active denervation to a region of the brachial triceps. In this case, MRI findings supported NA. Treatment-wise, the patient did not improve with anti-inflammatory and opioids, but her symptoms responded to a 27-day course of prednisone. On follow-up electrodiagnostic studies, denervation in the brachial triceps was absent. Zara et al.’s patient exhibited a highly focal and seemingly branch-specific presentation with overlapping clinical symptoms compared to our case [8]. Both instances demonstrate radial nerve branch involvement, a rarer finding in NA, highlighting the potential role of immune-mediated fascicular damage. 

Differential diagnoses

The focal presentation of NA necessitates consideration of differential diagnoses, including mononeuritis multiplex and compressive neuropathies, each with distinct clinical and diagnostic features.

Mononeuritis multiplex

Mononeuritis multiplex, a heterogeneous group of peripheral nerve disorders often associated with systemic vasculitis (e.g., polyarteritis nodosa or eosinophilic granulomatosis with polyangiitis), presents with asymmetric, multifocal sensory and motor peripheral nerve involvement due to ischemic damage [17]. Unlike NA, mononeuritis multiplex typically involves sensory deficits alongside motor symptoms in at least two peripheral or cranial nerves, and systemic symptoms such as fever, weight loss, or organ involvement are common [17]. In our case, the absence of sensory loss, systemic symptoms, or vasculitic markers (e.g., elevated erythrocyte sedimentation rate or antineutrophil cytoplasmic antibodies) favored NA. Diagnostically, nerve conduction studies (NCS) in mononeuritis multiplex may show axonal loss in multiple nerve distributions, whereas NA often shows focal denervation on EMG with normal sensory NCS [1,17]. Prognostically, mononeuritis multiplex carries a poorer outcome as the disease progression advances to become symmetrical and mimics distal dying-back symmetrical polyneuropathy over time.

Compressive neuropathies

No single guideline comprehensively covers all compressive neuropathies. Overall, carpal tunnel syndrome (CTS) dominates the literature due to its high prevalence. Compressive neuropathies, such as radial tunnel syndrome or isolated pectoral nerve compression, can mimic NA’s focal motor deficits. For example, radial tunnel syndrome may affect the posterior interosseous nerve, causing finger extensor weakness without sensory loss, but pain is typically located in the proximal forearm rather than the shoulder girdle, which is a more prevalent presentation of NA [18]. 

Similarly, isolated lateral and medial pectoral nerve compression or entrapment, as reported in weight lifters, presents with pectoralis major weakness and atrophy but may lack the painful prodrome seen in NA [19]. Nerve conduction findings in these cases revealed prolonged motor latency and reduced amplitude of the medial pectoral nerves and chronic changes without active denervation on EMG. Diagnostically, compressive neuropathies show focal conduction block on NCS at the compression site. In contrast, NA may or may not exhibit active denervation patterns on EMG despite normal sensory studies. However, differentiating these conditions can be complicated by potentially painless prodromes in NA and the lack of specific EMG findings [5]. Prognostically, compressive neuropathies may resolve with decompression, while NA recovery is slower and less predictable but generally spontaneous [5,19].

In our case, diagnosis was confounded by a pre-existing history of cervical myofascial pain previously responsive to trigger point injection, and the subsequent painful deep tissue massage, which the patient attributed to be the cause of his weakness and paresthesia, but may have simply been a further insult. When taking into account the full history of acute prodromal shoulder girdle pain, initial paresthesia, active focal denervation on EMG with associated atrophy, and limited disease progression followed by eventual spontaneous recovery, NA is the most likely diagnosis. 

Diagnostic and prognostic considerations

Differentiating NA from these conditions relies on clinical history, EMG/NCS, and imaging. The acute pain-prodrome-weakness sequence, normal sensory NCS, and patchy, focal denervation on EMG are hallmarks of NA [1]. High-resolution MRI or ultrasound, identifying hourglass-like constrictions, has been reported to enhance diagnostic specificity [6]. Prognostically, NA’s recovery is largely gradual and variable; overall, NA recovery is reported as ~80-90% of previous health after two to three years, though >70% are left with some residual paresis and exercise intolerance, especially of the periscapular muscles [5,10]. In contrast, mononeuritis multiplex and compression neuropathies require specific treatments (e.g., immunosuppression or steroids) to prevent progression, while compressive neuropathies may resolve with surgical intervention [17,18]. Our patient’s isolated lateral pectoral neuropathy and single triceps head atrophy suggest a favorable prognosis, given the limited extent of involvement. Still, long-term follow-up is warranted to monitor for residual deficits. In the context of emerging research, physical therapy focusing on motor control, energy conservation, and self-management techniques (pain, fatigue) rather than strength training appears to be a promising approach to long-term recovery [14]. 

Limitations

This study has multiple limitations. A case report lacks generalizability due to the rarity of the conditions selected for reporting. There is also no direct cause-and-effect relationship that can be established, in addition to a lack of a controlled comparative. It should also be addressed that patients’ cases are selected for publication due to the uniqueness of the encounter, and thus can be over-interpreted in the report. On the other hand, the strengths of this report include the educational value provided by sharing a unique pathology as well as the in-depth discussion of the diagnostic process for other physicians to recall if they encounter it in their practice.

## Conclusions

Our case of NA with isolated lateral pectoral neuropathy and single triceps head atrophy exemplifies the condition’s selective involvement of less common peripheral nerves and distal branches, consistent with other reports of isolated involvement. Compared to the broader involvement in other reports, the focal nature of our case highlights NA’s variable presentation and the diagnostic value of natural history and EMG. Careful exclusion of differential diagnoses like mononeuritis multiplex, compressive, infectious, or inflammatory neuropathies is crucial, based on clinical, laboratory, electrodiagnostic, and imaging findings. This atypical presentation underscores the need for prompt, comprehensive work-up and tailored management in NA, further adding to the literature on the diverse spectrum of clinical presentations. 
